# The Science of Meteorology, Etc.

**Published:** 1872-09

**Authors:** Thomas D. King

**Affiliations:** Montreal


*The Science of Meteorology—Its Utility—The Necessary Instruments, and How to Use Them.* By Mr. THOMAS D. KING, Montreal.

It cannot have escaped the attention of those whose acquirements enable them to judge, and who have the opportunities of examining the state of Meteorological Science in England and the United States, that in Canada, more particularly in the Province of Quebec, with respect to the simplest weather reports, the amount of rain-fall and the multitude of causes by which the atmosphere is influenced either for the benefit or destruction of animal and vegetable life, we are almost in a state of ignorance.

That a city like Montreal, eminently distinguished for its commerce, for its manufactories, and for its philanthrophy, should be indifferent to the progress of inquiries which are so necessary; and that the medical faculty should be dependent upon the observations of persons whose reports are published at their own discretion, is a fact which is well deserving the attention of those who shall inquire into the causes that influence the scientific progress of the neighboring States. In them are Observatories and Scientific Institutions founded and endowed by private citizens.

and supplemented by grants from Congress, for the discovery of new truths, and for the diffusion of these among men. There is also a large staff of private observers, as well as those belonging to the U. S. Army Corps, in all many hundreds, who contribute to Meteorological Science, serving to place in a clear point of view, the connection of climate with the natural productions of different parts of the earth.

That the state of knowledge in any country will exert a directive influence on the general system of instruction adopted in it, is a principle too obvious to require investigation. And it is equally certain that the tastes and pursuits of our manhood will bear on them the traces of the earlier impressions of our education. It is not therefore unreasonable to suppose that the neglect of science, we may almost say, the utter neglect of science, in the Province of Quebec, may be attributed to the system of education we pursue.

Young men pass away from our Public Schools, Colleges, and Universities, ignorant almost of every branch of useful knowledge, more particularly of the application of science to Arts and Manufactures. Our system of education may be attributed in some measure to the fact that amongst the wealthy and middle classes, scientific knowledge scarcely exists. Those who have chosen the profession of medicine, may have a slight knowledge of Chemistry, Zoology, Botany, Vegetable Physiology, and Mineralogy, but they rarely possess a knowledge of those physical laws which brought forth a Dalton, a Davy, a Faraday—nevertheless, there is a knowledge which they ought to possess, viz.: The peculiarities of the atmosphere which affect the functions of organized bodies. It would be productive of useful results, if physicians of extensive practice, more particularly those attached to our Infirmaries and Hospitals, would make accurate meteorological registers, especially during the prevalence of any epidemic or contagious disorders; not that it must be considered that atmospheric peculiarities alone produce epidemic and other complaints, which must be regarded as having a compound origin, and as resulting from the operation of peculiar states of atmosphere on persons of particular states of constitution; otherwise all persons would be affected, which is contrary to experience. Of this there can be no doubt, that the effect of atmospheric changes upon ordinary diseases requires more attention from medical men than it has hitherto received.

My object is not to write a treatise on meteorology; that task has been done by many able men. Those who have the leisure to go deeply into the subject, and wish to become acquainted with scientific meteorology, will find that the following works exhaust the topic, viz.:

Luke Howard, "Climate of London."
Daniell, "Meteorological Essays."

Kaemtz, "Meteorology."
Drew, "Practical Meteorology."
Glaisher, "Hygrometrical Tables."
James, "Instructions for Taking Meteorological Observations."
Fitz-roy, "Weather Book."
Proceedings of the Meteorological Society.
Journal of the Scottish Meteorological Society.
Symons, "British Rain-fall," and "Meteorological Magazine."
Steinmetz, "Manual of Weathercasts" and "Sunshine and Showers."
Smithsonian Institution Publications.
"Directions for Meteorological Observations and the Registry of Periodical Phenomena."
"Tables, Meteorological and Physical," prepared by Arnold Guyot, Professor of Geology and Physical Geography, College of New Jersey.

To the pages of some of these I am indebted chiefly for the few concluding remarks I shall deem necessary to make. The subject is an important one, and at the same time for all practical purposes very simple. It requires no overwhelming attention; the observer does not require to be well acquainted with mathematics or astronomy; he does not require, like some ambitious of scientific distinction, to have attached to his name a kind of comet, carrying with it a tail of letters, such as LL.D., D.C.L., F.R.S., F.M.B.S., F.L.S., etc.; the observations demand no unrivaled accuracy, but mere ordinary care, such as would be required by a schoolboy in a common sum of vulgar fractions.

My object is not to satisfy the requisitions of Science, but to induce those who have not hitherto paid any attention to the subject to do so at once, and to form, if possible, in the Province of Quebec, a Meteorological Society, for the purpose of collecting observations on the weather, the amount of rain-fall, and the registry of periodical phenomena which can be published monthly in this journal.

To the readers of this journal it is not necessary to say that an acquaintance with the science of Meteorology, together with the observance of instrumental and natural signs of the changes and conditions of the atmosphere about us, enable the formation of a foreknowledge of the kind of weather, as of storms, excess of heat or cold, drought or rain. To seamen, fishermen, farmers, gardeners, builders, engineers, travelers, more than the generality of people, such foreknowledge is of great value, on account of their pursuits being greatly affected by changes in the weather. Indeed, the personal safety and comfort of everybody, in a greater or less degree, must be promoted by the ability to prognosticate the extremes of the weather.

It is now well known that variations in the intensity and duration of sunshine, the exposure to humidity, and the amount and frequency of rain and snow, have highly important influences upon the development and growth of crops. A farmer would, therefore, undoubtedly acquire increased experience and knowledge of the varied operations of his calling if he were to register weather observations upon a simple, but uniform plan, noting all the signs afforded by nature. The blights which affect vegetation, such as the mildew and smut of wheat; the fungus, which attacks the vine; the fly, which destroys the hop and the turnip, may all be dependent upon atmospheric conditions, which attentive observations may detect.

It will now be necessary to say a few words about

METEOROLOGICAL INSTRUMENTS, AND HOW TO USE THEM.

The instruments absolutely required for prognosticating the weather, are few in number, and such as need very little practice to secure accurate and useful information. Meteorological investigators must be cautioned against the so-called "cheap" instruments paraded in the shops, as utterly useless and likely to disgust them with the science. It is certain that no one need be without good, reliable instruments on account of the cost. The best instrument-makers in England seem all anxious to meet the views of those who wish to devote a portion of their time to the development of meteorology; and one of them, Mr. Pastorelli, of Piccadilly, London, has designed a complete set of instruments at a very moderate cost, the barometer having been examined by Mr. Glaisher, of the Greenwich Observatory, the well-known meteorological authority, and certified to read correctly to .01 or .02 with the standard, and the remainder of the instruments verified at the Observatory of Greenwich.

A set of the out-of-door instruments, fixed on a neat stand, would form an ornament to any lawn or grass plot near the house, and one of our daughters or sons might undertake the daily inspection of the instruments and keep the register of the weather. Fathers of families have thus the means of introducing into their homes a new feature of interest, and even of utility; for there would thus be in every family a reliable weather-prophet, whose timely advice might prevent exposure to many a drenching, and to the damage and health of garments. Excursions would not, if the Barometer and Hygrometer were consulted, be undertaken in the utter uncertainty as to the weather that is likely to attend them. Besides, it is no small satisfaction to be able to know when we are likely to need umbrellas, and when we may leave the encumbrance at home although the sky be overcast and cloudy. This degree of certainty is within the reach of all of us, with the aid of the requisite instruments.

In all public schools such a set of instruments should form part of the daily study; and we have no doubt that our young ones would take to it as kindly as to any other pursuit likely to excite their natural curiosity.

I shall now proceed to notice succinctly the various instruments required in the investigation of the weather.

### THE BAROMETER.

This instrument indicates the changes in the weight, or rather the elasticity of the air; the more elastic the air, the finer the weather, and the higher the barometer. The elasticity of the air being diminished, the barometer falls proportionately to the disturbance causing bad weather.

In fixing the barometer, select a position commanding a good light, but not exposed to sunshine, and adjust the tube to a vertical position by means of a plumb-line. Before reading the instrument, tap it gently a few times, as the mercury is apt to adhere to the tube. In reading, let the eye be placed on the exact level of the mercurial column, so that the eye, the back and forepart of the index, and the top of the column, be in the same horizontal plane.

In forming a judgment of forthcoming weather, the point at which the mercury stands should not be so much regarded as whether it is rising or falling; and much consideration should habitually be given to its movements during the previous two or three days. Different latitudes and elevations above the sea have their peculiar *par-line*, or average height of the barometric column.

The varying pressure or elasticity of the air, as shown by the barometer's rise and fall, must have some specific influence on the public health, the *fall* being attended with aggravation or production of diseases of an inflammatory or hæmorrhagic character, the rise producing or aggravating those of a plethoric character, such as pulmonary and cerebral apoplexy, congestive bronchitis, etc. On the other hand, increased atmospheric pressure exercises a sedative influence on the respiration and pulse, diminishing the frequency, but generally increasing the force of both. The lungs are more fully expanded, the blood is more completely oxygenated, and the nervous and digestive organs acquire increased vigor—as forcibly shown by the vigor of our "jolly tars," whose life is passed "on the ocean wave" or "the sea-level."

### THE THERMOMETER.

The intelligent observation of the thermometer should always accompany that of the barometer.

The thermometer should be placed so as to be freely exposed to the surrounding air, and protected from the effects of reflected heat, radiation, and rain. The instruments should always be read when we read the barometer.

DRY BULB THERMOMETER.

This and all out-door thermometers should be read to tenths of degrees; it is very easy to estimate these parts of a degree. Always look square at a thermometer, or you will read it too high or too low; read it as quickly as practicable, and don't breathe upon it.

WET BULB THERMOMETER.

Muslin to be kept clean, changed every month or so. The water used should be either clean rain or distilled. Hard water deposits salts on the bulb and hardens the muslin. In frosty weather the bulb should be wetted with water at about 45 degrees, fifteen minutes before observing.

MAXIMUM THERMOMETER.

Whether this be constructed on the plan patented by Negretti or on that of Professor Phillips, it is to be used in a horizontal position; the reading of the end of the column furthest from the bulb is the maximum, and the instrument is reset by lowering the bulb end; sometimes they require a more or less smart shake—of course, gentle means should be tried first.

MINIMUM THERMOMETER.

There is at present nothing better than Pastorelli's spirit thermometer for general use; the position of the end of the index furthest from the bulb is the reading to be entered, and the instrument is to be set by raising the bulb so that the index may fall to the end of the column.

Thermometers should be constructed so as to be without errors, or to have errors less than 0.5 of a degree, which may be neglected for ordinary purposes, and applied when using the observations for scientific purposes.

HYGROMETER, OR DRY AND WET BULB THERMOMETER.

The bulb of the wet thermometer is covered with thin muslin, round the neck of which is twisted a conducting thread of lamp-wick, common darning cotton, or floss silk; this passes into an adjacent vessel of water placed at such a distance as to allow a length of conducting thread of about three inches. The cup or glass should be placed on one side and a little beneath, so that the water within may not affect the reading of the dry bulb by its too near vicinity.

The importance of this instrument to the requirments of a sick chamber are scarcely to be over-rated, and will be at once obvious to all who know that the comfort of the patient is dependent not so much on the temperature, as on the hygrometric condition of the air. In our long winters the air of the apartment when heated with stoves, is not unfrequently too dry, in which case the difference between the readings of the two thermometers will be great, and this condition will be manifest to the sufferer by the degree of inconvenience he will experience attributable to this cause. If the air be moist, the difference between the readings will be less in proportion to the degree of moisture; and if the air be saturated, the readings will be alike. *It would be well for the medical profession to enforce, as far as lies in its power, the use of this simple and effectual instrument, which at all times is valuable with reference to the record of external temperature, as well as hygrometric conditions of the air, and which in case of sickness gives indications so important to the comfort and convalescence of the patient.*

If the hygrometer shows increasing dampness by the difference of the readings becoming *smaller*, then *rain* may be expected; but if the hygrometer shows continuing or increasing dryness, by the reverse, then we may expect more wind, without rain.

The dryness or humidity of the air has the greatest influence on the development of diseases, and therefore this instrument should be the test of the climate of places to which invalids are sent for the recovery of health. In the open sea the air seems to be in a state of saturation, and the quantity of vapor is greatest on coasts, diminishing as we approach the interior.

The hygrometer should be in universal use in our changeable climate, not only during hay-making and the all-important time of harvest, but also to do away with the many doubts about the weather from the mere appearances of the sky, causing the great inconvenience of carrying umbrellas, when not likely to be required.

### RAIN GAUGE.

This instrument informs us of the quantity of rain that has fallen at any given time. There is a great variety of forms, but perhaps the best and the simplest is Howard's Rain Gauge.

The rain-fall throughout Canada is very varied in its amount in different localities, depending upon the peculiar features of the country.

The following table shows the equivalent of rain in inches, its weight per acre, and bulk in gallons:

| | | | | | | | | | | |
|---|---|---|---|---|---|---|---|---|---|---|
| Inches of rain.................... .. | 0·1 | 0·2 | 0·3 | 0·4 | 0·5 | 0·6 | 0·7 | 0·8 | 0·9 | 1 in. |
| Tons, per acre.......................... | 10 | 20 | 30 | 40 | 50 | 61 | 71 | 81 | 91 | 101 |
| Gallons, per acre...................... | 2262 | 4525 | 6787 | 9049 | 11312 | 13574 | 15836 | 18098 | 20361 | 22623 |

The following fact will give some idea of the quantity of rain that falls in showers, and the amount of human labor by hand-irrigation that would be required for an equivalent. Suppose a flower garden 22 yards square (a tenth of an acre,) how many cans of water are required to equal *half an inch* of rain—a very moderate amount for a thunder-shower?

If the can holds four gallons (i.e. 40 lbs.) of water, it will require to be filled 282 times, or, to put it in weight, 5 tons of water would have to be supplied.

WIND DIRECTION.

Observers should not rely upon weathercocks for the direction of the wind. It is better to watch the way clouds are drifting; they are steadier in their course than vanes, flags, streamers, or even smoke, driven by the surface wind. Moreover, weathercocks are sometimes set incorrectly; either the variation of the compass has not been allowed for, or it has been applied the wrong way.

The meteorological instruments required for ordinary observations in the pursuit of weather wisdom are few:—Barometer, Hygrometer, Self-Registering Thermometers, Rain Gauge.

A little practice will render the duty quite easy, and enable the observer ere long to judge for himself concerning coming weather. To doubt that science of weather is possible, would be to doubt that atmospheric disturbances are governed by fixed laws. But, indeed, a wonderful change has taken place in this respect of late years. Formerly most *savans* scoffed at the idea of predicting the weather; and Arago, the French astronomer, said that no scientific man, anxious for his reputation, would venture upon such a thing even for the space of a single day. But now it is just the reverse; those most acquainted with meteorology are the staunchest believers in the ultimate probability of "doing the thing" to the world's great satisfaction. Indeed, had one-half of the time and research devoted to sidereal astronomy been spent in observing and registering changes which any one may notice, but which no one has yet succeeded in predicting or interpreting, meteorology would not be still "in its infancy" after a birth thousands of years ago, nay, coeval with the first appearance of man upon earth, to observe "the signs which are in the firmament of the heavens."—*Canada Med. & Surg. Journal.*

